# CircMAPK1 induces cell pyroptosis in sepsis-induced lung injury by mediating KDM2B mRNA decay to epigenetically regulate WNK1

**DOI:** 10.1186/s10020-024-00932-6

**Published:** 2024-09-19

**Authors:** Min Li, Hanjing Lu, Chujun Ruan, Qiao Ke, Longhui Hu, Zhao Li, Xiaoran Liu

**Affiliations:** 1 Emergency Department of Hainan General Hospital (Hainan Affiliated Hospital of Hainan Medical University), Haikou, 570311 Hainan Province China; 2 Emergency trauma College of Hainan Medical University, Haikou, 571199 Hainan Province China; 3 The First Affiliated Hospital of Hainan Medical College, Key Laboratory of Emergency and Trauma of Ministry of Education, No.3 Xueyuan Road, Longhua District, Haikou, 571199 Hainan Province China

**Keywords:** CircMAPK1, Macrophage pyroptosis, Sepsis, KDM2B, WNK1

## Abstract

**Background:**

Macrophage pyroptosis is a pivotal inflammatory mechanism in sepsis-induced lung injury, however, the underlying mechanisms remain inadequately elucidated.

**Methods:**

Lipopolysaccharides (LPS)/adenosine triphosphate (ATP)-stimulated macrophages and cecal ligation and puncture (CLP)-induced mouse model for sepsis were established. The levels of key molecules were examined by qRT-PCR, Western blotting, immunohistochemistry (IHC) and ELISA assay. The subcellular localization of circMAPK1 was detected by RNA fluorescence in situ hybridization (FISH). Cell viability, LDH release and caspase-1 activity were monitored by CCK-8, LDH assays, and flow cytometry. The bindings between KDM2B/H3K36me2 and WNK1 promoter was detected by chromatin immunoprecipitation (ChIP) assay and luciferase assay, and associations among circMAPK1, UPF1 and KDM2B mRNA were assessed by RNA pull-down or RNA immunoprecipitation (RIP) assays. The pathological injury of lung tissues was evaluated by lung wet/dry weight ratio and hematoxylin and eosin (H&E) staining.

**Results:**

CircMAPK1 was elevated in patients with septic lung injury. Knockdown of circMAPK1 protected against LPS/ATP-impaired cell viability and macrophage pyroptosis via WNK1/NLRP3 axis. Mechanistically, loss of circMAPK1 enhanced the association between KDM2B and WNK1 promoter to promote the demethylation of WNK1 and increase its expression. CircMAPK1 facilitated KDM2B mRNA decay by recruiting UPF1. Functional experiments showed that silencing of KDM2B or WNK1 counteracted circMAPK1 knockdown-suppressed macrophage pyroptosis. In addition, silencing of circMAPK1 alleviated CLP-induced lung injury in mice via KDM2B/WNK1/NLRP3 axis.

**Conclusion:**

CircMAPK1 exacerbates sepsis-induced lung injury by destabilizing KDM2B mRNA to suppress WNK1 expression, thus facilitating NLRP3-driven macrophage pyroptosis.

**Supplementary Information:**

The online version contains supplementary material available at 10.1186/s10020-024-00932-6.

## Introduction

Sepsis, predominantly caused by bacterial infections, manifests as a severe systemic inflammatory response syndrome (SIRS) (Wang and Liu 2023; Patel et al. 2022; Singer et al. 2016). Acute lung injury (ALI) is a notable complication of sepsis which characterized by intense lung inflammation, significant hypoxemia and decreased lung compliance (Sadowitz et al. 2011; Liu et al. 2022a, b; Reiss et al. 2012). Deciphering the mechanism by which sepsis induces ALI is vital for progress in medical and biological research.

In ALI, recruited monocytes differentiate into macrophages in the lungs, where they can undergo pyroptosis which is a pro-inflammatory form of cell death (Cheng et al. 2021; Wei et al. 2022a, b). Caspase activation-mediated pyroptosis exacerbates lung inflammation and tissue damage by releasing inflammatory cytokines and cellular contents, thus contributing to the progression of sepsis-induced ALI (Jiao et al. 2021; Liu et al. 2022a, b). It is well-accepted that activated NLPR3 inflammasome activates pro-caspase-1, causing the release of inflammatory cytokines IL-1β and IL-18, and caspase-1 also cleaves GSDMD into GSDMD-N and triggers pyroptosis (Shi et al. 2015). Recent studies have illustrated that inhibition of NLRP3-mediated pyroptosis protects against sepsis-induced ALI (Liu et al. 2022a, b; Shi et al. 2022). However, the specific mechanism underlying macrophage pyroptosis in sepsis-induced ALI remains unclear.

Emerging evidence supports that circular RNAs (circRNAs) regulate gene expression in sepsis (Niu et al. 2022), and their unique structure and expression patterns offer potential as promising biomarkers and therapeutic targets in sepsis (Wei et al. 2022a, b). RNA sequencing has identified a number of differentially expressed circRNAs in pulmonary macrophages, and these circRNAs may be implicated in the regulation of apoptosis, inflammation and mitochondria distribution in sepsis-induced ALI (Bao et al. 2019). However, the detailed mechanism by which the dysregulated circRNAs contribute to the pathogenesis of sepsis-induced ALI merits in-depth investigation. Our preliminary data have showed that circMAPK1 (hsa_circ_0008870, mmu_circ_0006095) was significantly upregulated in peripheral blood mononuclear cells (PBMCs) of septic lung injury patients by transcriptome sequencing (Fig. S1). This finding led us to hypothesize that the aberrant expression of circMAPK1 may influence macrophage pyroptosis in the lungs. The objective of this study is to explore the potential role of circMAPK1, particularly its impact on macrophage pyroptosis in ALI.

RNA-binding proteins (RBPs) function as pivotal conduits in the cascade of circRNA downstream regulatory pathways (Zang et al. 2020). These proteins regulates the molecular stability and subcellular distribution circRNA, as well as their interactions with target RNA molecules (Zheng et al. 2021; Zhang et al. 2022). Upstream frameshift 1 (UPF1) has emerged as a notable candidate for its potential binding affinity to circMAPK1 and KDM2B mRNA (RPISeq). Importantly, UPF1 plays a crucial role in the regulation of mRNA stability and degradation processes (Staszewski et al. 2023). Although UPF1 has not yet been explored in sepsis, previous studies have described the association of UPF1 with inflammatory responses in lung ischemia/reperfusion injury (Gao et al. 2023), raising the possibility that UPF1 may regulate inflammation in sepsis-induced lung injury. UPF1 potentially functions as a RBP for circMAPK1, thereby modulating KDM2B expression.

WNK lysine deficient protein kinase 1 (WNK1), a kinase that regulates intracellular ion homeostasis, plays a significant role in controlling macrophage pyroptosis (Zhao et al. 2022). Recent study has reported that WNK1 is a negative regulator of NLRP3-mediated macrophage pyroptosis (Mayes-Hopfinger et al. 2021). Despite this known function, the specific mechanism of WNK1 in NLRP3-mediated macrophage pyroptosis and its role in sepsis-induced lung injury remains unelucidated. Our analysis based on MethPrimer suggests that the WNK1 promoter region contains methylated CpG islands. This finding indicates that hypermethylation in this region may lead to reduced WNK1 expression, potentially triggering macrophage pyroptosis. Lysine-specific demethylase 2B (KDM2B, also known as FBXL10), a histone demethylase targeting H3 histone, plays a pivotal role in regulating gene expression (Zheng et al. 2018). It has been reported that KDM2B occupies nearly all CpG-dense promoters to prevent promoter hypermethylation (Boulard et al. 2015). Additionally, the recruitment of KDM2B to gene promoters is a key factor in modulating gene expression (Vargas-Ayala et al. 2019). The histone mark H3K36me2, influenced by KDM2B, also contributes to shaping the DNA methylation landscape across intergenic regions (Weinberg et al. 2019). However, the specific role of KDM2B in potentially mediating the hypermethylation of WNK1 in sepsis-induced ALI remains an area for further investigation.

In summary, we hypothesized that circMAPK1 facilitated KDM2B mRNA decay via recruiting UPF1, and the inhibition of KDM2B-mediated WNK1 demethylation further suppressed WNK1 expression, thus triggering NLRP3-mediated pyroptosis in sepsis-induced ALI. This study lies in its contribution to the nuanced understanding of the molecular interactions in sepsis, and circMAPK1/KDM2B/WNK1/NLRP3 axis was identified as a promising target for the targeted therapeutic strategy of sepsis-induced ALI.

## Methods

### Clinical sample collection

Whole blood samples were collected from 40 septic patients and 40 healthy volunteers. Adult patients who were diagnosed with sepsis in the ICU based on Sepsis-3 criteria, excluding those with chronic infections or immunosuppression, were recruited to this study. This study protocol was reviewed and approved by IBR of Hainan Medical University, No. HYLL-2021-392 and informed consent were obtained. Blood collection was performed using sterile venipuncture technique.

### Primary bone marrow-derived macrophages (BMDMs) isolation

Primary BMDMs were isolated from C57BL/6J mice (Hunan SJA Laboratory Animal Co. Ltd., Changsha, China) as previously described (Lin et al. 2023). Briefly, mice were euthanized, and the bone marrow was harvested from both tibias and femurs by flushing the bone cavities with ice-cold PBS. This marrow extract was filtered through a 100-µm nylon mesh, followed by the centrifugation at 300 g for 10 min. BMDMs were then cultured in DMEM (Gibco, Grand Island, NY, USA) containing 10% FBS (Gibco), and 30 ng/mL recombinant murine M-CSF (Gibco) at 37 °C/5% CO_2_.

### Cell culture, treatment and transfection

Mouse macrophage cells line RAW264.7 cells were from ATCC (Manassas, VA, USA). Primary BMDMs and RAW264.7 cells were cultured in DMEM containing 10% FBS, and incubated at 37 °C in a 5% CO_2_ atmosphere. Both RAW264.7 and BMDMs were exposed to Lipopolysaccharide (LPS, 100 ng/mL, Sigma-Aldrich, St. Louis, MO, USA) and ATP (5 mM, Sigma-Aldrich) for 6 h to induce pyroptosis. For knockdown experiments, shRNAs targeting circMAPK1, KDM2B, and WNK1 were designed and cloned into a pLKO.1 (Sigma-Aldrich). The sequence of circMAPK1 was cloned into a pcDNA3.1 vector (Invitrogen, Carlsbad, CA, USA). Primary BMDMs and RAW264.7 cells were transfected with overexpression construct or shRNA using Lipofectamine 3000 (Invitrogen).

### Characterization of circMAPK1

Total RNA was extracted using the miRNeasy Mini Kit (Qiagen), and 2 µg RNA was subjected to RNase R treatment (6 U/3 µL, Epicentre, WI, USA) at 37 °C. To assess RNA stability, RAW264.7 cells were treated with Actinomycin D (5 µg/mL, Sigma-Aldrich) for 0, 4, 8, 12, 16–20 h. The expression of MAPK1 or circMAPK1 was quantified by qRT-PCR. The sequence of circMAPK1 was confirmed by Sanger sequencing.

### Subcellular fractionation

Nuclear and cytoplasmic RNAs were extracted from RAW264.7 cells using PARIS Kit (Invitrogen). In brief, RAW264.7 cells were harvested and resuspended in Cell Fractionation Buffer. The cytoplasmic fraction (supernatant) was collected after centrifugation, and nuclear pellet was then lysed with Cell Disruption Buffer. RNA was extracted from nuclear and cytoplasmic fractions following the manufacturer’s protocol. The expression circMAPK1 was detected by qRT-PCR.

### RNA fluorescence in situ hybridization (FISH)/immunofluorescence (IF)

RAW264.7 cells were fixed with 4% paraformaldehyde (PFA) and permeabilized with 0.5% Triton X-100. RAW264.7 cells were then hybridized with Cy3-labeled circMAPK1, U6 or 18 S FISH probe (GenePharma, Shanghai, China) at 37 °C overnight. For RNA FISH/IF staining, the fixed and permeabilized slides were hybridized with Cy3-labeled circMAPK1, followed by the overnight staining of anti-F4/80 antibody (1:50, 14-4801-82, Invitrogen). The slides were then incubated with anti-mouse secondary antibody-Alexa Fluor 488 (1:500, A-11029, Invitrogen). Nucleus was visualized by DAPI (Invitrogen), and the slides were examined under a confocal microscope (Nikon, Tokyo, Japan).

### Quantitative real-time polymerase chain reaction (qRT-PCR)

Total RNA was extracted from blood samples, macrophages or tissues using Trizol LS or Trizol reagent (Invitrogen). cDNA was synthesized using SuperScript IV Reverse Transcriptase (Invitrogen), and qRT-PCR was conducted in ABI7500 Real-Time System using SYBR Green MasterMix (Thermo Fisher Scientific). Relative gene expression was calculated using 2^−ΔΔCT^ method with GAPDH or U6 used as an internal control.

### Methylation-specific PCR (MSP)

The methylation of the WNK1 promoter was determined by MSP using specific primers. PCR reaction was performed using bisulfite-treated DNA as a template, and PCR products were detected by 2% agarose gel electrophoresis.

### Enzyme-linked immunosorbent assay (ELISA)

The serum levels of IL-1β and IL-18 were assessed using commercial human ELISA kits (IL-18, ab215539, Abcam and IL-1β, ab214025, Abcam). The levels of IL-1β and IL-18 in the cell culture media were detected using commercial mouse ELISA kits (IL-18, ab216165, Abcam and IL-1β, ab197742, Abcam). Briefly, whole blood samples were allowed to clot for 30 min, and serum was collected after centrifugation. Cell culture supernatant was harvested and centrifuged at 1500 g for 10 min. ELISA assay was conducted following the manufacture’s instruction. A450 was measured using a BioTek microplate reader.

### Cell counting kit-8 (CCK-8) assay

BMDMs and RAW264.7 cells (5,000 cells/well) were seeded in a 96-well plate. At 6 h post-treatment, 10 µL of CCK-8 solution (Dojindo, Kumamoto, Japan) was added to each well. After 1 h incubation, A450 was measured using a microplate reader (BioTek).

### Lactate dehydrogenase (LDH) analysis

BMDMs and RAW264.7 cells were seeded in a 96-well plate. At 6 h post-treatment, the culture media were collected and analyzed using LDH Cytotoxicity Assay Kit (Beyotime, Shanghai, China) following the manufacture’s protocol. A490 was measured using a microplate reader (BioTek).

### FAM-FLCA caspase assay

BMDMs and RAW264.7 cells were stained with FAM-YVAD-FMK (Immunochemistry, Davis, CA, USA) at 37 °C for 1 h. This was followed by the fixation with Fixative (#636, Immunochemistry) and PI staining. The stained cells were then subjected to flow cytometry analysis using BD FACSAria Flow Cytometer (Franklin Lakes, NJ, USA).

### Chromatin immunoprecipitation (ChIP) assay

ChIP assay was conducted using Pierce Agarose ChIP Kit (26156, Pierce). Briefly, BMDMs and RAW264.7 cells were cross-linked with 1% formaldehyde and lysed. The chromatin fragments were prepared by MNase digestion and incubated with normal IgG or antibody against H3K36me2 (2 µg, MA5-14867, Invitrogen), KDM2B (2 µg, 17-10264, Millipore, Billerica, MA, USA) at 4 °C overnight. The DNA/protein complexes were then enriched by Protein A/G agarose. DNA was purified and analyzed by qRT-PCR.

### Dual luciferase reporter assay

The promoter region of WNK1 was cloned into pGL-3 vector (Promega, Madison, WI, USA). shNC/shcircMAPK1/shcircMAPK1 + shKDM2B and luciferase reporter construct were co-transfected into RAW264.7 cells and BMDMs, and relative luciferase activity was measure at 48 h post-transfection using Dual Luciferase Reporter System (Promega). Renilla luciferase was used as an internal control.

### RNA pull-down assay

RNA pull-down assay was performed using Pierce RNA Pull-Down Kit (20164, Pierce). In brief, a biotinylated probe recognized the back-splice junction of circMAPK1 was conjugated to streptavidin magnetic beads. This was followed by the incubation with cell lysates. The enriched RNA/protein complexes were then eluted and analyzed by Western blotting using anti-UPF1 antibody. A scrambled probe was used as a negative control.

### RNA immunoprecipitation (RIP) assay

RIP assay was conducted using Magna RIP Kit (17–700, Millipore). Briefly, cells were lysed with RIP Lysis Buffer. Antibody against UPF1 (1:20, ab109363, Abcam) or normal rabbit IgG was conjugated to Protein A/G beads, followed by the incubation with cell lysates at 4 °C overnight. RNA was then purified, and the level of circMAPK1 was detected by qRT-PCR.

### Cecal ligation and puncture (CLP) model

Male C57BL/6 mice (8 ∼ 12-week-old, 20 ∼ 25 g, *n* = 6 per group) were obtained from Hunan SJA Laboratory Animal Co. Ltd. (Changsha, China). These mice were anesthetized with isoflurane. A midline abdominal incision was made to expose the cecum, which was then partially ligated with a suture and punctured with a sterile needle (21–23 gauge). After the procedure, the abdomen was closed with surgical sutures post-procedure, and mice received post-operative care including fluid resuscitation with saline and analgesics with buprenorphine. Sham mice were subjected to the same procedure without ligation and puncture treatments (Rittirsch et al. 2009). For the in vivo knockdown of circMAPK1, mice were administered with AAV9-shcircMAPK1 (SyngenTech, Beijing, China) or AAV9-shNC via the tail vein.

### Histological analysis

Lung tissues were fixed with 10% formalin and embedded in paraffin, and sectioned into thin slices using a microtome. The slides were stained with hematoxylin and eosin (H&E) solution (Sigma-Aldrich). Under a microscope, inflammatory cell infiltration, alveolar wall thickening, vascular congestion, hemorrhage, and epithelial cell damage were assessed and scored on a scale from 0 to 4 for an overall lung injury assessment (Li et al. 2019).

### Lung wet/dry weight ratio

The wet/dry weight ratio of the lung was calculated by measuring the weight of the left lung before and following a 70 h drying period at 24 °C (Zhang et al. 2014).

### Immunohistochemistry (IHC) and IF analysis

The sections were deparaffinized and rehydrated. Antigen retrieval was carried out using a citrate buffer. After blocking with 1% BSA, the sections were then incubated with primary antibodies against F4/80 (1:50, 14-4801-82, Invitrogen), MPO (1:200, ab208670, Abcam) or caspase-1 (1:500, ab138483, Abcam), followed by the incubation with HRP, Alexa Fluor 555- or Alexa Fluor 488-conjugated secondary antibody (Invitrogen). For IHC analysis, detection was achieved using a 3,3’-Diaminobenzidine substrate (Vector Laboratories, CA, USA) for visualization. The sections were counterstained with hematoxylin (Sigma-Aldrich) or DAPI to highlight the nuclei.

### Terminal deoxynucleotidyl transferase dUTP nick end labeling (TUNEL)

Tissues were fixed with formaldehyde (Sigma-Aldrich), then embedded in paraffin. The tissues were sectioned at 5 μm. Permeabilization was performed using proteinase K (Roche, Basel, Switzerland). The sections were incubated with TUNEL reaction mixture containing terminal deoxynucleotidyl transferase (TdT) and biotin-labeled nucleotides (Thermo). After incubation, the labeled DNA was visualized using streptavidin-HRP and DAB substrate. Images were acquired using a microscope (Nikon).

### Western blot analysis

Proteins were lysed with RIPA lysis buffer (Pierce, Rockford, IL, USA) and separated by gel electrophoresis. Proteins were then transferred onto a PVDF membrane (Pierce). The blots were incubated with primary antibody at 4 °C overnight after blocking. This was followed by the incubation with HRP-conjugated secondary antibody (Invitrogen). Signals were visualized using a chemiluminescence detection system (Pierce). Primary antibodies used in Western blotting: anti-KDM2B (1:500, 65999, Proteintech, Wuhan, China), anti-WNK1 (1:1000, MA5-35466, Invitrogen), anti-NLRP3 (1:250, MA5-23919, Invitrogen), anti-caspase-1 (1:1000, ab138483, Abcam), anti-ASC (1:1000, 04-147, Sigma-Aldrich), anti-GSDMD-N antibody (1:1000, #10137, CST, Danvers, MA, USA), anti-H3K36me2 antibody (1:1000, MA5-14867, Invitrogen) and anti-UPF1 (1:5000, 66898-1-Ig, Proteintech) antibodies.

### Statistical analysis

Data was presented in the Mean ± SD format, with a minimum of three replicates per experimental condition. We conducted data analysis using GraphPad Prism 8.0, utilizing unpaired t-tests for comparisons between groups and one-way ANOVA with *Turkey post-hoc test* for multiple group comparisons. Significance was determined based on the calculated *P*-values, with a threshold of *P* < 0.05 considered statistically significant.

## Results

### CircMAPK1 is upregulated in patients with sepsis-induced lung injury

**Fig. 1:**
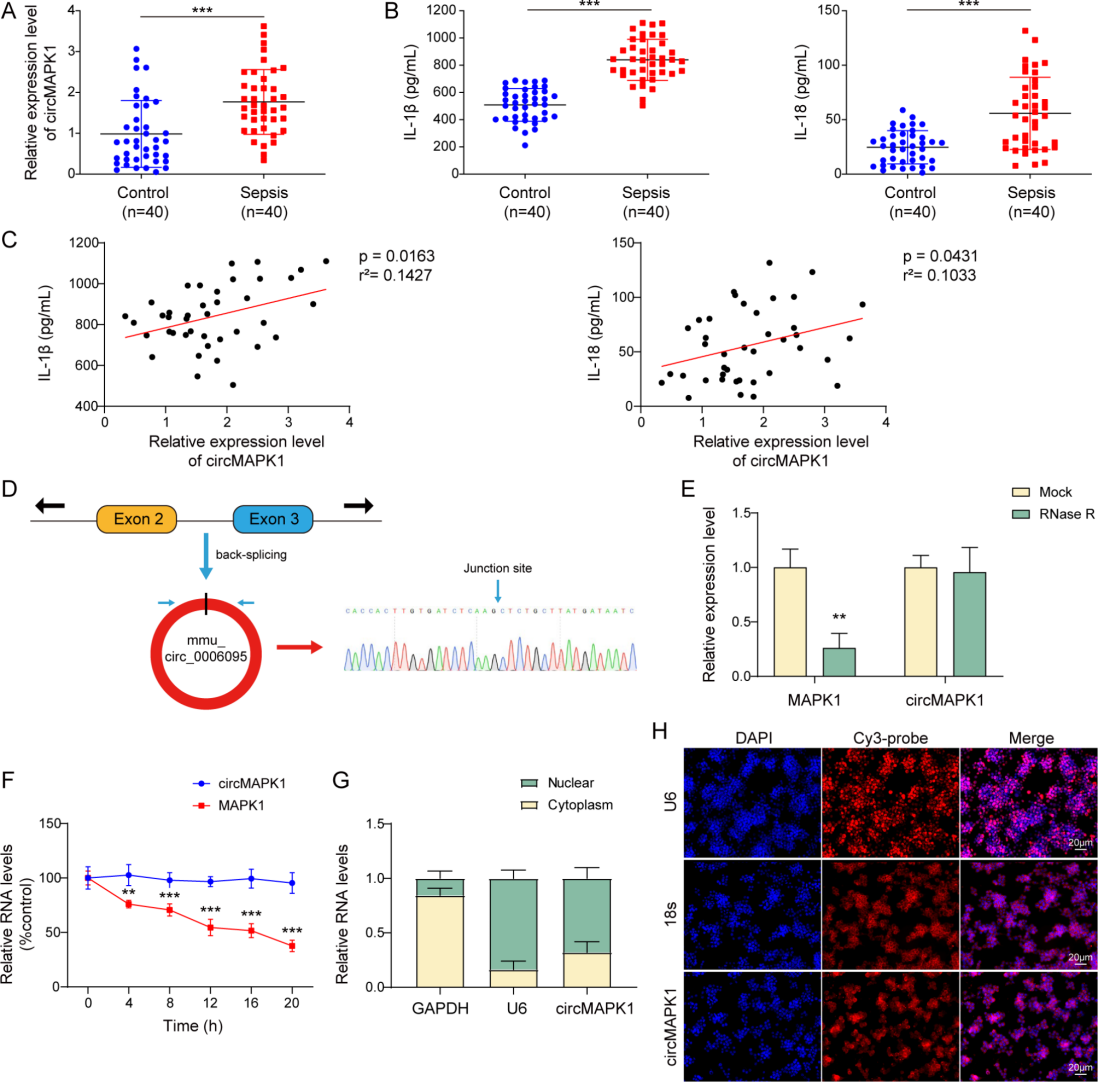
CircMAPK1 is upregulated in sepsis-induced lung injury patients. (**A**) qRT-PCR was used to measure circMAPK1 expression in sepsis-induced lung injury patients. (**B**) ELISA assay was employed to quantify IL-1β and IL-18 levels in the serum of sepsis-induced lung injury patients. (**C**) Pearson correlation analysis assessed the relationship between circMAPK1 levels and IL-1β as well as IL-18 levels in sepsis-induced lung injury patients. (**D**) circMAPK1 was assessed by Sanger sequencing. (**E&F**) circMAPK1 stability was examined using qRT-PCR after Actinomycin D and RNase R treatments. (**G&H**) The subcellular localization of circMAPK1 was determined through subcellular fractionation and qRT-PCR, as well as RNA FISH. Red, circMAPK1; Blue, DAPI. Scale bar, 20 μm. *P < 0.05, **P < 0.01, ***P < 0.001

We sought to explored the role of circMAPK1 in t sepsis-induced lung injury. A significant upregulation of circMAPK1 was observed in patients suffering from sepsis-induced lung injury (Fig. 1A), along with elevated serum levels of the inflammatory cytokines IL-1β and IL-18 (Fig. 1B). In addition, Pearson correlation analysis showed a positive correlation between circMAPK1 and IL-1β/IL-18 levels in patients with sepsis-induced lung injury (Fig. 1C), suggesting the potential role of circMAPK1 in modulating inflammation in sepsis-induced lung injury. Furthermore, detailed molecular characterization of circMAPK1 was carried out. circMAPK1 was originated from the 2nd to 3rd exons of the MAPK1 gene on mouse chromosome 16 with 373 nt in length (Fig. 1D). RNA stability assay revealed that circMAPK1 degraded time-dependently in the presence of transcription inhibitor Actinomycin D, and the degradation of circMAPK1 was much slower than that of MAPK1 upon Actinomycin D or RNase R treatment (Fig. 1E&F). Subcellular fractionation and RNA FISH unequivocally showed that the predominant localization of circMAPK1 in the cytoplasm of macrophages (Fig. 1G&H). These findings present evidence of the potential role of circMAPK1 in the inflammatory process of sepsis-induced lung injury.

### Silencing of circMAPK1 inhibits NLRP3-mediated macrophage pyroptosis

**Fig. 2:**
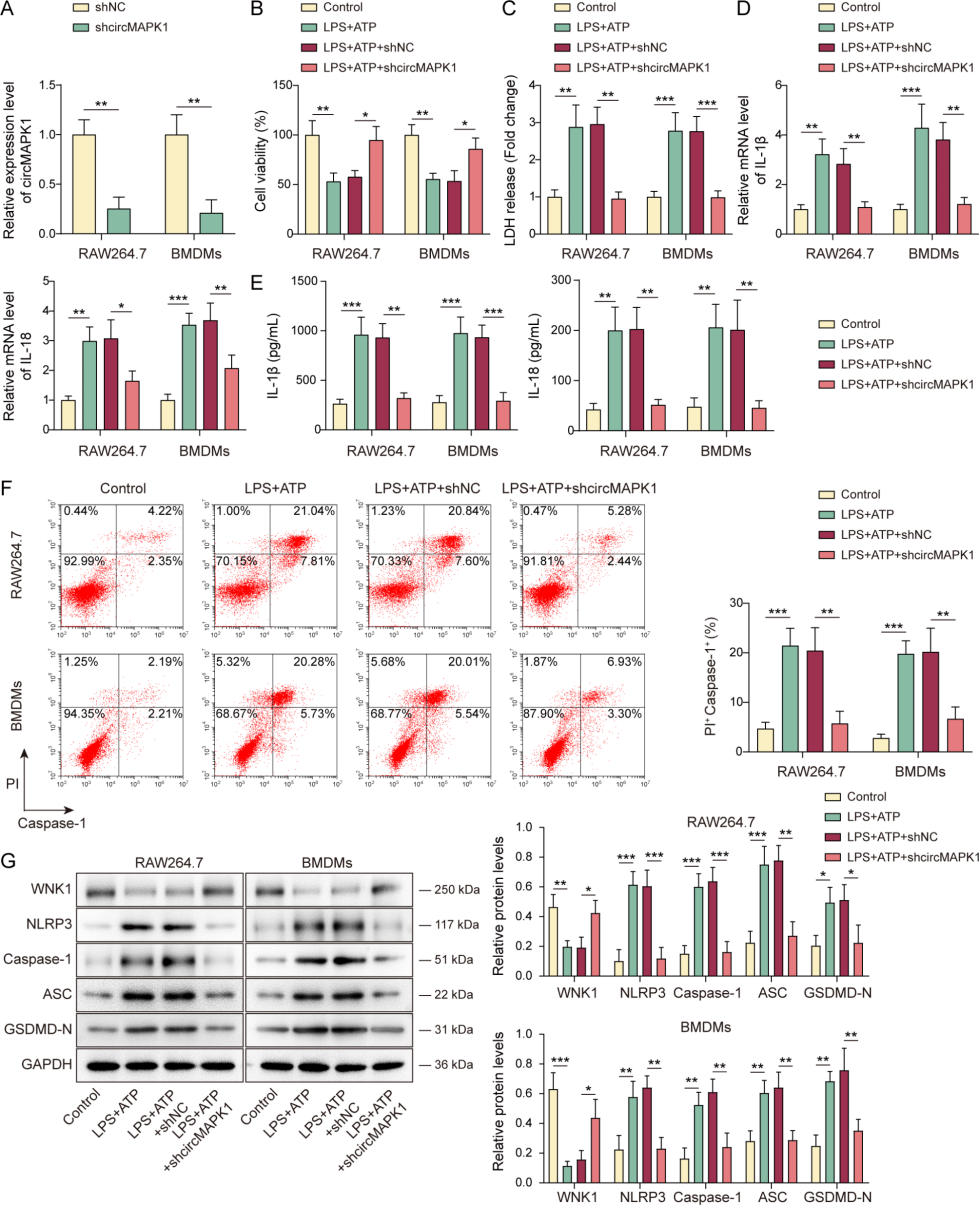
Silencing of circMAPK1 inhibits NLRP3-mediated macrophage pyroptosis. RAW264.7 cells and BMDMs were transfected with shNC or shcircMAPK1. (**A**) qRT-PCR was employed to assess circMAPK1 levels in macrophages. Transfected macrophages were exposed to LPS/ATP treatments. (**B&C**) Cell viability and LDH release levels were determined by CCK-8 and LDH assays, respectively. (**D&E**) The expression and secretion of IL-1β and IL-18 were measured by qRT-PCR and ELISA assay, respectively. (**F**) Caspase-1 activity in macrophages was evaluated by flow cytometry. (**G**) The protein levels of WNK1, NLRP3, caspase-1, ASC and GSDMD-N in macrophages were detected by Western blotting with quantitative analysis. *P < 0.05, **P < 0.01, ***P < 0.001

Since NLRP3-mediated macrophage pyroptosis is closely related to the inflammatory process of sepsis-induced lung injury (Liu et al. 2022a, b), our study aimed to investigate whether circMAPK1 contributed to the inflammation in sepsis-induced lung injury by modulating macrophage pyroptosis. In this study, RAW264.7 cells and BMDMs were transfected with a shRNA targeting circMAPK1 (Fig. 2A) and subsequently exposed to 100 ng/mL LPS and 5 mM ATP for 6 h to induce pyroptosis. CCK-8 and LDH release assays revealed that knockdown of circMAPK1 significantly mitigated the LPS/ATP-mediated reduction in cell viability and the elevation of LDH level (Fig. 2B&C). Moreover, silencing of circMAPK1 reversed LPS/ATP-induced IL-1β and IL-18 expression and secretion in RAW264.7 cells and BMDMs, respectively (Fig. 2D&E). Flow cytometry further showed that circMAPK1 knockdown rescued LPS/ATP-mediated increase of caspase-1 activity in macrophages (Fig. 2F). Additionally, circMAPK1 knockdown counteracted LPS/ATP-induced changes of key proteins involved in NLRP3-mediated pyroptosis, including NLRP3, caspase-1, ASC and GSDMD-N, as well as LPS/ATP-decreased WNK1 (Fig. 2G). These findings highlight the critical role of circMAPK1 in regulating NLRP3-mediated macrophage pyroptosis in sepsis-induced lung injury.

### CircMAPK1 silencing enhances WNK1 expression through KDM2B-mediated WNK1 demethylation

**Fig. 3:**
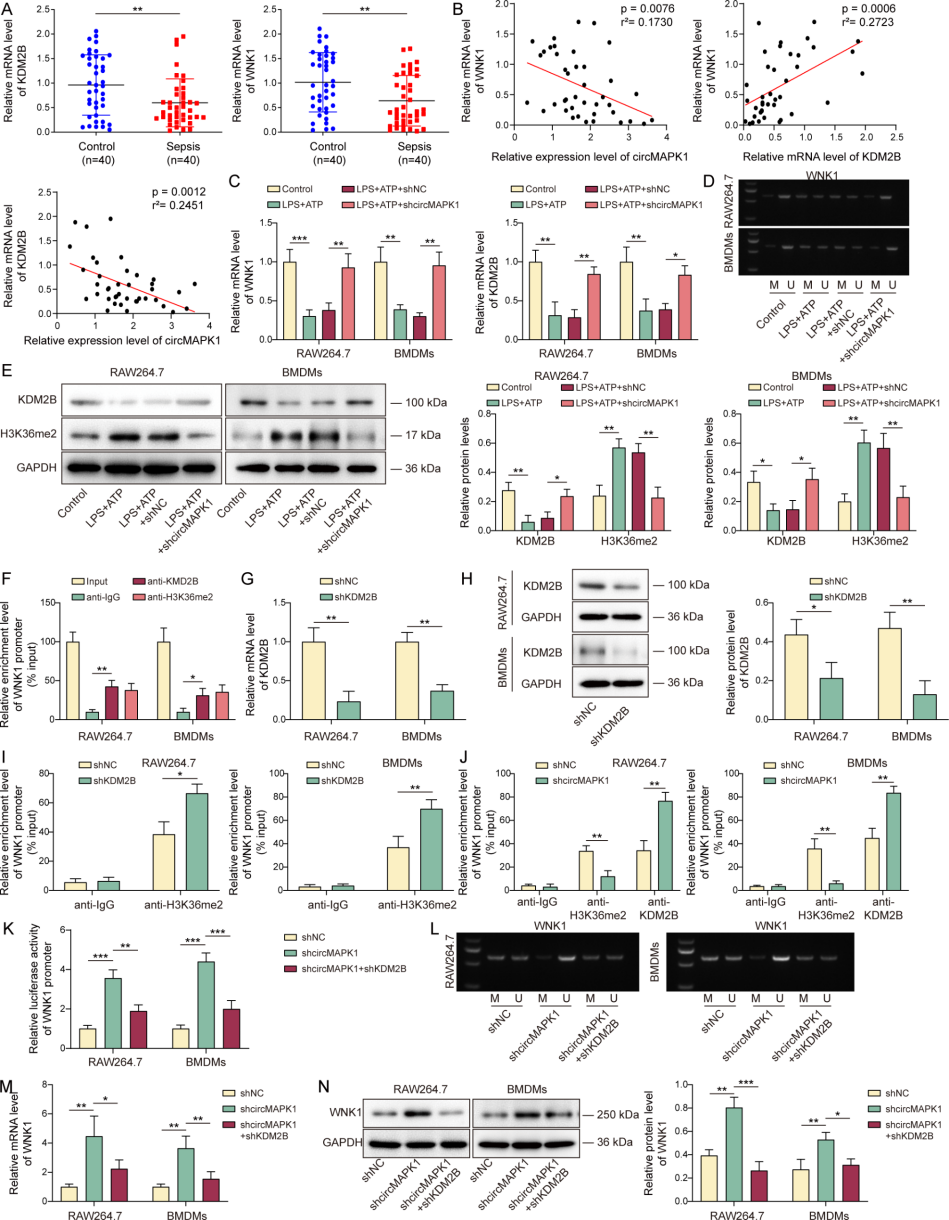
CircMAPK1 silencing enhances WNK1 expression through KDM2B-mediated WNK1 demethylation. (**A**) The mRNA levels of KDM2B and WNK1 in the blood samples of patients with sepsis-induced lung injury were detected by qRT-PCR. (**B**) Pearson correlation analysis was conducted to evaluate the correlations among circMAPK1, WNK1 and KDM2B in patients with sepsis-induced lung injury. Macrophages were transfected with shNC or shcircMAPK1, followed by the treatments of LPS/ATP. (**C**) The mRNA levels of WNK1 and KDM2B in macrophages were detected by qRT-PCR. (**D**) The methylation of WNK1 promoter was detected by MSP assay. (**E**) The protein levels of KDM2B and H3K36me2 in macrophages were detected by Western blotting with quantitative analysis. (**F**) The associations between KDM2B/H3K36me2 and WNK1 promoter were assessed by ChIP assay. Macrophages were transfected with shNC or shKDM2B. (**G&H**) The mRNA and protein levels of KDM2B in macrophages were detected by qRT-PCR and Western blotting, respectively. (**I**) The interaction between H3K36me2 and WNK1 promoter was detected by ChIP assay in macrophages were transfected with shKDM2B. Normal IgG served as a negative control. (**J**) The interaction between H3K36me2 and WNK1 promoter was detected by ChIP assay in macrophages were transfected with shcircMAPK1. Normal IgG served as a negative control. Macrophages were transfected with shNC, shcircMAPK1 or/and shKDM2B. (**K**) The luciferase activity was measured using dual luciferase reporter system. (**L**) The methylation of WNK1 promoter was detected by MSP assay. (**M&N**) The mRNA and protein levels of WNK1 in macrophages were detected by qRT-PCR and Western blotting, respectively. *P < 0.05, **P < 0.01, ***P < 0.001

WNK1 has been identified as a negative regulator of NLRP3-mediated macrophage pyroptosis (Mayes-Hopfinger et al. 2021), raising the possibility that WNK1 may be involved in circMAPK1-regulated macrophage pyroptosis. To further understand the mechanism underlying circMAPK1/WNK1 axis in sepsis-induced lung injury, we first examined the expression of KDM2B and WNK1 in septic patients. As presented in Fig. 3A, KDM2B and WNK1 were markedly decreased in the blood samples of patients with sepsis-induced lung injury, compared with that of healthy volunteers. There was a negative correlation between circMAPK1 and WNK1, as well as between circMAPK1 and KDM2B, while WNK1 positively correlated with KDM2B in patients with sepsis-induced lung injury (Fig. 3B). Silencing of circMAPK1 led to rebounds of WNK1 and KDM2B in RAW264.7 cells and BMDMs (Fig. 3C). Subsequent MSP assay revealed a high degree of methylation on WNK1 promoter upon LPS/ATS stimulation in macrophages, whereas knockdown of circMAPK1 decreased the methylation of WNK1 promoter (Fig. 3D). Given that KDM2B modulates DNA methylation and it acts as a specific demethylase for H3K36me2 (He et al. 2011), KDM2B and H3K36me2 expression were examined in macrophages. As expected, LPS/ATP-downregulated KDM2B and LPS/ATP-upregulated H3K36me2 were reversed by circMAPK1 knockdown. (Fig. 3E). ChIP assay further revealed the enrichments of KDM2B and H3K36me2 at WNK1 promoter (Fig. 3F). Knockdown study showed that transfection of shKDM2B successfully decreased KDM2B expression (Fig. 3G&H), and lack of KDM2B enhanced the enrichment of H3K36me2 at WNK1 promoter (Fig. 3I). Similarly, silencing of circMAPK1 increased the enrichment of KDM2B at WNK1 promoter, but decreased the association between H3K36me2 and WNK1 promoter in both RAW264.7 cells and BMDMs (Fig. 3J). Consistently, luciferase reporter assay showed that lack of circMAPK1 induced WNK1 promoter activity, while KDM2B knockdown further attenuated shcircMAPK1-increased WNK1 promoter activity in RAW264.7 cells and BMDMs (Fig. 3K). shcircMAPK1-decreased WNK1 methylation was reversed by KDM2B knockdown in macrophages (Fig. 3L). Loss of circMAPK1 upregulated WNK1 expression, while KDM2B knockdown counteracted shcircMAPK1-induced WNK1 mRNA and protein levels in macrophages (Fig. 3M&N). These findings suggest that lack of circMAPK1 increases WNK1 expression via KDM2B-mediated WNK1 demethylation.

### CircMAPK1 facilitates KDM2B mRNA decay by recruiting UPF1

**Fig. 4:**
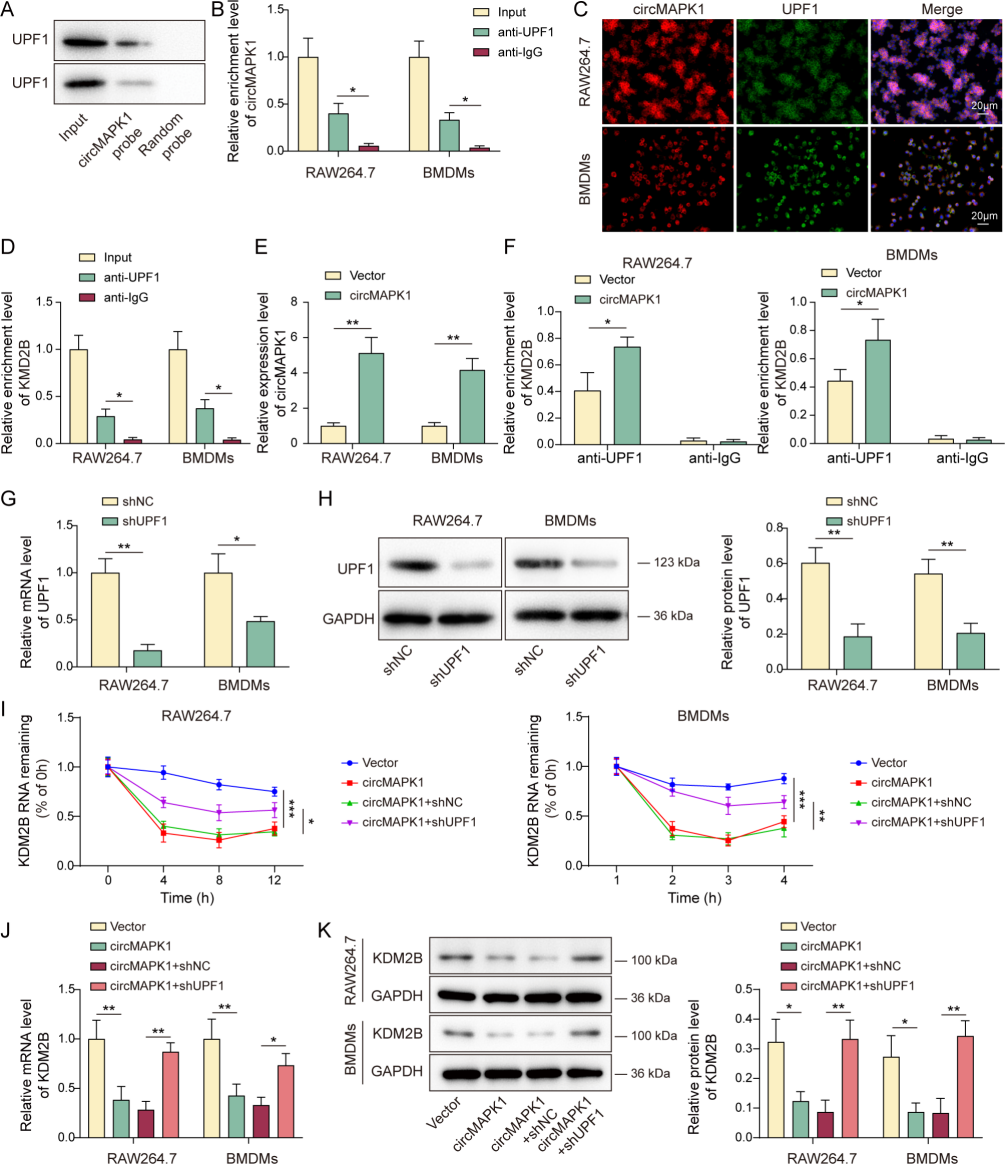
CircMAPK1 facilitates KDM2B mRNA decay by recruiting UPF1. (**A&B**) RNA pull-down and RIP assays were conducted to investigate the interaction between circMAPK1 and UPF1. (**C**) RNA FISH and immunofluorescent staining were used to examine the co-localization of circMAPK1 and UPF1 in macrophages. Red, circMAPK1; Green, UPF1. Scale bar, 20 μm. (**D**) RIP assay were employed to detect the binding of UPF1 to KDM2B mRNA. Macrophages were transfected with circMAPK1 overexpression vector. (**E**) The level of circMAPK1 in macrophages was detected by qRT-PCR. (**F**) RIP assay was utilized to assess the association between UPF1 and KDM2B mRNA. Macrophages were transfected with shNC or shUPF1. (**G&H**) The mRNA and protein levels of UPF1 were detected by qRT-PCR and Western blotting. Macrophages were co-transfected with circMAPK1 overexpression construct and shNC/shUPF1. (**I**) The mRNA stability of KDM2B in macrophages were detected by qRT-PCR in the presence of Actinomycin D. (**J&K**) The mRNA and protein levels of KDM2B were detected by qRT-PCR and Western blotting. *P < 0.05, **P < 0.01, ***P < 0.001

To elucidate the mechanism by which circMAPK1 modulated KDM2B expression, we leveraged the RPISeq Database (http://pridb.gdcb.iastate.edu/RPISeq/) to identify RNA-binding proteins (RBPs) associated with KDM2B and circMAPK1, pinpointing UPF1 as a potential interactor. RNA pull-down assay showed that biotinylated circMAPK1 probe successfully pulled down UPF1 in both RAW264.7 cells and BMDMs (Fig. 4A). Conversely, antibody against UPF1 immunoprecipitated circMAPK1 in macrophages (Fig. 4B). RNA FISH also revealed the co-localization of circMAPK1 and UPF1 in RAW264.7 cells and BMDMs (Fig. 4C). Moreover, a direct association between UPF1 and KDM2B mRNA was also detected by RIP assay (Fig. 4D). Notably, overexpression of circMAPK1 enhanced the interaction between UPF1 and KDM2B mRNA in macrophages (Fig. 4E&F). Additionally, transfection of shUPF1 successfully decreased UPF1 expression in both RAW264.7 cells and BMDMs (Fig. 4G&H). RNA stability assay further showed that circMAPK1 overexpression promoted KDM2B mRNA decay, while silencing of UPF1 reversed circMAPK1-impaired mRNA stability of KDM2B in macrophages (Fig. 4I). Furthermore, circMAPK1 overexpression decreased KDM2B mRNA and protein levels in macrophage, whereas UPF1 knockdown counteracted these effects (Fig. 4J&K). These data suggest that circMAPK1 promotes KDM2B mRNA decay by recruiting UPF1.

### CircMAPK1 triggers macrophage pyroptosis through the KDM2B/WNK1/NLRP3 pathway

**Fig. 5:**
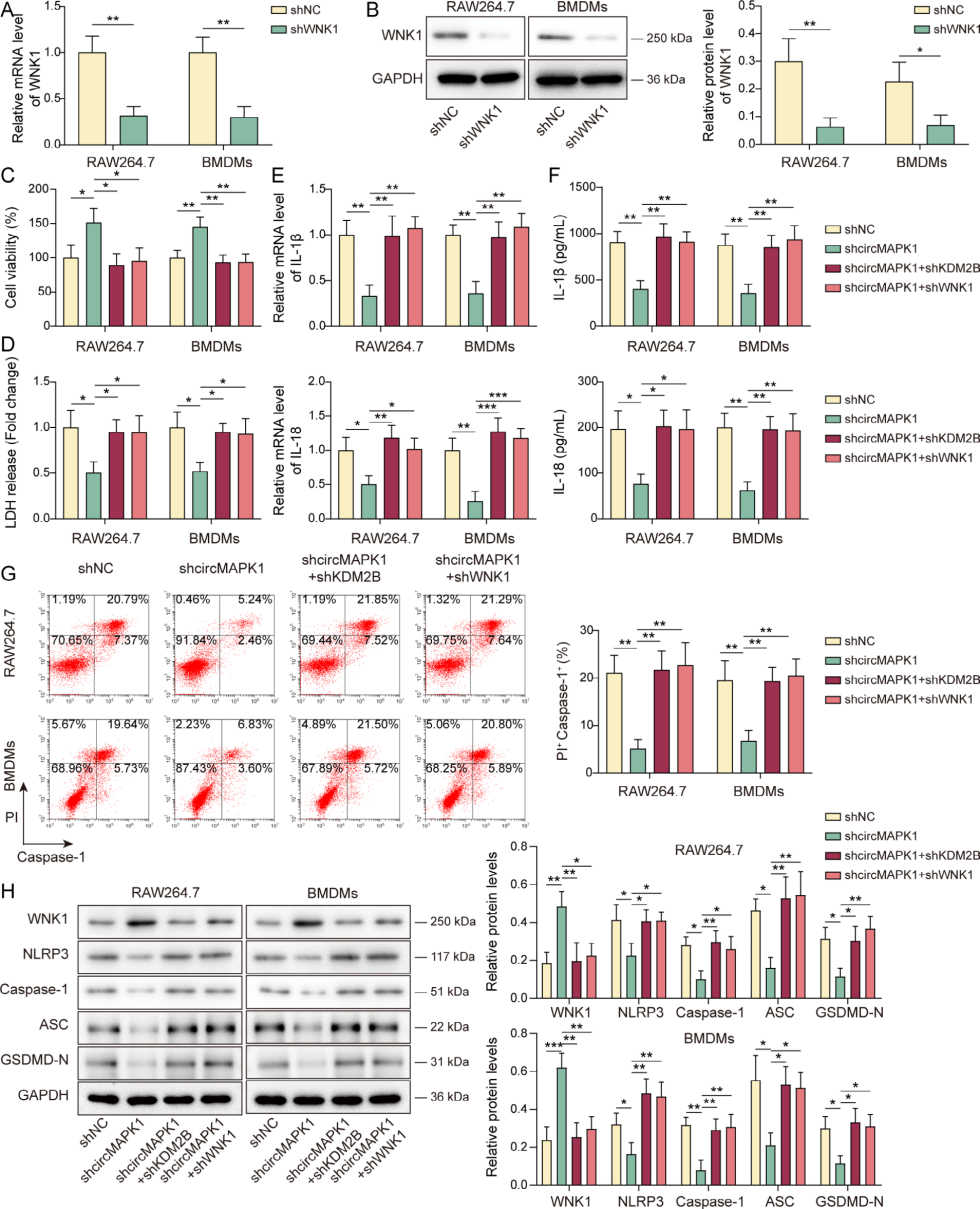
CircMAPK1 triggers macrophage pyroptosis through the KDM2B/WNK1/NLRP3 pathway. Macrophages were transfected with shNC/shWNK1, followed by LPS/ATP stimulation. (**A&B**) The mRNA and protein levels of WNK1 in macrophages were detected by qRT-PCR and Western blotting. Macrophages were transfected with shcircMAPK1 or/and shKDM2B/shWNK1. (**C&D**) Cell viability and LDH release levels were measured using CCK-8 and LDH assays, respectively. (**E&F**) The expression and secretion of IL-1β and IL-18 in macrophages were determined by qRT-PCR and ELISA assay, respectively. (**G**) Caspase-1 activity was analyzed by flow cytometry. (**H**) The protein levels of WNK1, NLRP3, caspase-1, ASC and GSDMD-N in macrophages were detected by Western blotting with quantitative analysis. *P < 0.05, **P < 0.01, ***P < 0.001

Functional experiments were next conducted to test if silencing of KDM2B or WNK1 mitigated the effects of circMAPK1 downregulation in macrophages. As presented in Fig. 5A&B, knockdown of WNK1 markedly downregulated WNK1 mRNA and protein levels in macrophages. In addition, circMAPK1 knockdown-rescued cell viability and LDH release were counteracted by KDM2B or WNK1 knockdown (Fig. 5C&D). Similarly, shcircMAPK1-suppressed IL-1β and IL-18 expression and secretion, as well as shcircMAPK1-decreased caspase-1 activity, were rescued by KDM2B or WNK1 knockdown (Fig. 5E-G). Furthermore, shcircMAPK1-mediated upregulation of WNK1, and shcircMAPK1-downregulated NLRP3, caspase-1, ASC and GSDMD-N were reversed by shKDM2B and shWNK1 in RAW264.7 cells and BMDMs (Fig. 5H). These findings suggest that circMAPK1 regulates macrophage pyroptosis in sepsis-induced lung injury via KDM2B/WNK1/NLRP3 axis.

### CircMAPK1 downregulation alleviates lung injury in CLP mice

**Fig. 6:**
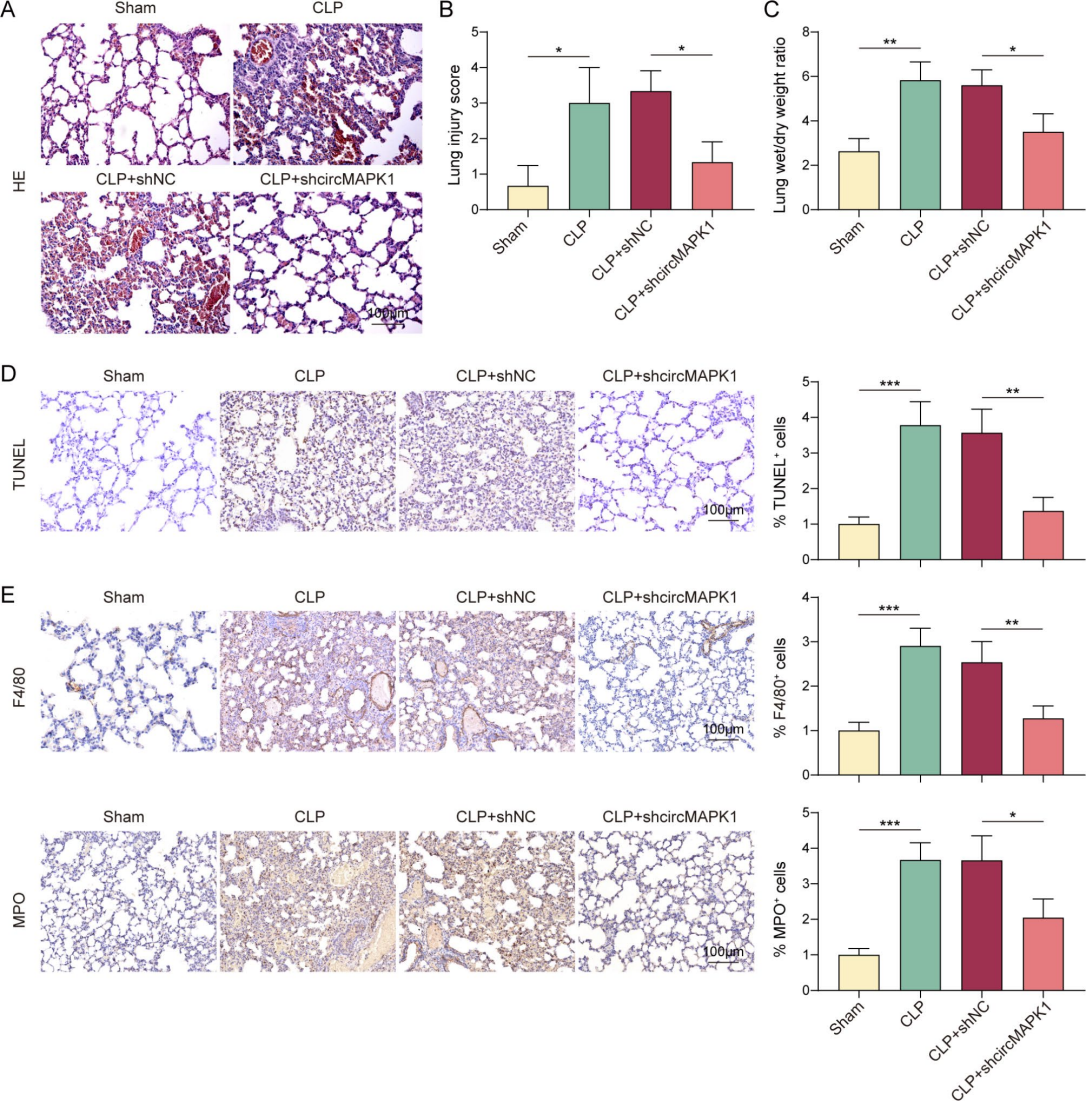
CircMAPK1 downregulation alleviates the lung injury in CLP mice. Mice were randomly divided into four groups (*n* = 6 per group): Sham, CLP, CLP + shNC; CLP + shcircMAPK1. (**A&B**) Representative images of H&E staining and statistical analysis of lung tissue injury scores. Scale bar, 100 μm. (**C**) The lung tissue wet-to-dry weight ratios with quantitative analysis. (**D**) Cell apoptosis in mouse lung tissues was assessed by TUNEL staining. Scale bar, 100 μm. (**E**) The immunoreactivities of F4/80 and MPO in lung tissues were detected by IHC staining with quantitative analysis. Scale bar, 100 μm. *P < 0.05, **P < 0.01, ***P < 0.001

To further investigated the function of circMAPK1 in vivo, a septic mouse model was established using CLP method (Dejager et al. 2011). H&E staining demonstrated significant disorganization and damage in lung tissues of CLP mice, which were notably mitigated by circMAPK1 silencing (Fig. 6A&B). circMAPK1 knockdown effectively protected against CLP-elevated lung wet-to-dry weight ratio which was recognized as a key indicator of pulmonary edema in mice (Fig. 6C). Additionally, cell apoptotic rates were much higher in lung tissues of CLP mice, but it decreased following circMAPK1 silencing (Fig. 6D). Furthermore, the upregulation of F4/80 and MPO in lung tissues of CLP mice, whereas these effects on F4/80 and MPO were reversed by circMAPK1 knockdown (Fig. 6E). These findings indicate that silencing of circMAPK1 ameliorates CLP-induced lung injury in vivo.

### Silencing of circMAPK1 inhibits macrophage pyroptosis in CLP mice through the KDM2B/WNK1/NLRP3 pathway

**Fig. 7:**
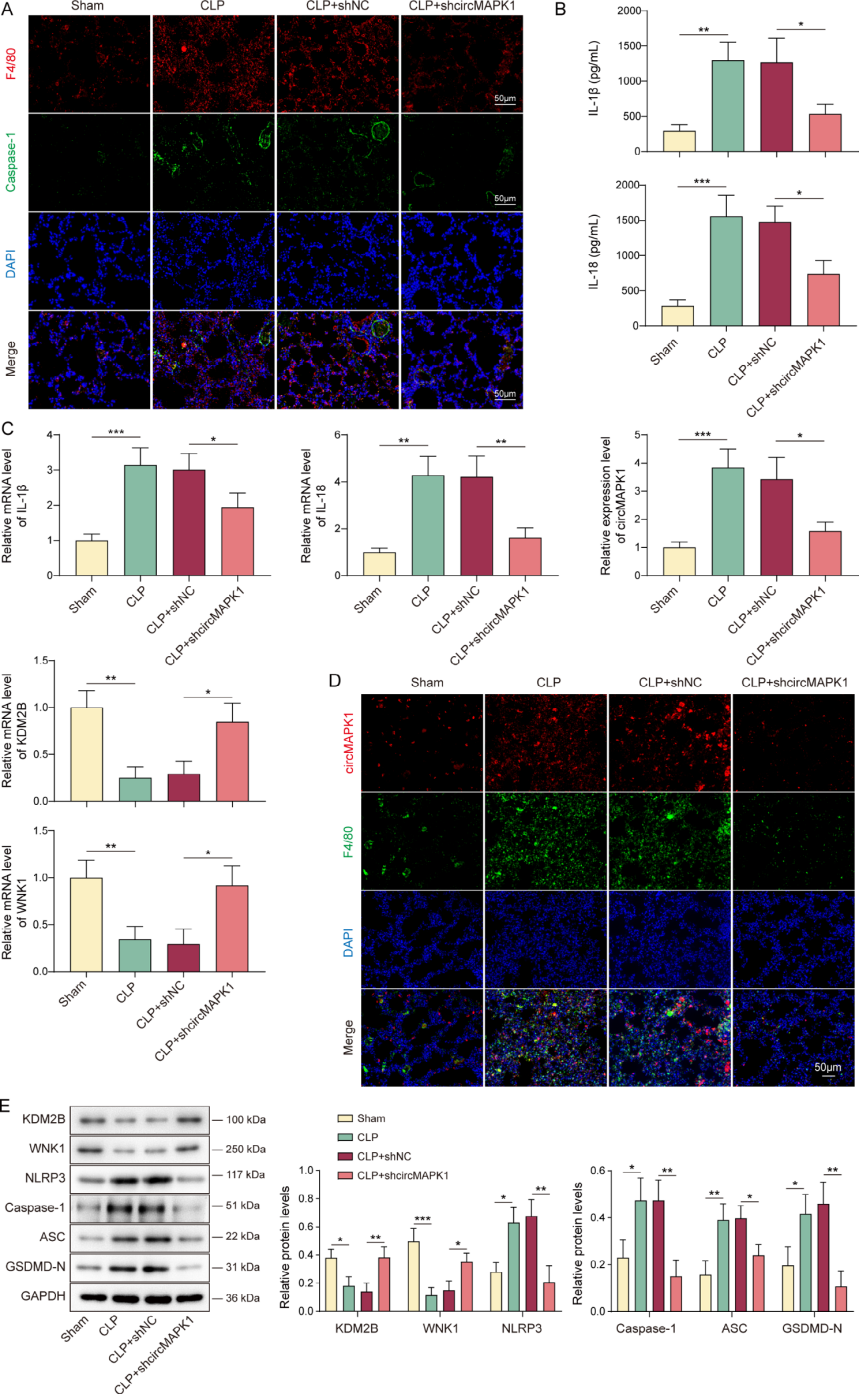
CircMAPK1 silencing inhibits macrophage pyroptosis in CLP mice through the KDM2B/WNK1/NLRP3 pathway. Mice were randomly divided into four groups (*n* = 6 per group): Sham, CLP, CLP + shNC; CLP + shcircMAPK1. (**A**) The immunoreactivities of F4/80 and caspase-1 in lung tissues were detected by IF staining. Red, F4/80; Green, caspase-1; Blue, DAPI. Scale bar, 50 μm. (**B**) The levels of IL-1β and IL-18 in lung tissues were quantified by ELISA assay. (**C**) qRT-PCR was employed to measure IL-1β, IL-18, circMAPK1, KDM2B, and WNK1 levels in mouse lung tissues. (**D**) The colocalization of circMAPK1 and F4/80 was detected by RNA FISH/IF staining. Red, circMAPK1; Green, F4/80. Scale bar, 50 μm. (**E**) The protein levels of KDM2B, WNK1, NLRP3, caspase-1, ASC and GSDMD-N in mouse lung tissues were detected by Western blotting with quantitative analysis. *P < 0.05, **P < 0.01, ***P < 0.001

In our advanced mechanistic study involving the CLP sepsis model, we found that F4/80-positive macrophages exhibited increased caspase-1 activity in lung tissues of CLP mice, while circMAPK1 silencing attenuated these effects (Fig. 7A). Moreover, CLP mice exhibited increased expression of IL-1β and IL-18 in lung tissues (Fig. 7B&C), along with the upregulation of circMAPK1 and downregulation of KDM2B and WNK1 (Fig. 7C). Notably, these alterations were reversed by circMAPK1 knockdown (Fig. 7B&C). In addition, RNA FISH/IF staining showed the co-localization of circMAPK1 and the macrophage marker F4/80 in CLP mouse lung tissues (Fig. 7D). Correspondingly, CLP reduced KDM2B and WNK1 expression and elevated NLRP3, caspase-1, ASC, and GSDMD-N in mouse lung tissues, all of which were counteracted by circMAPK1 knockdown (Fig. 7E). These findings indicate that circMAPK1 regulates macrophage pyroptosis via the KDM2B/WNK1/NLRP3 pathway.

## Discussion

NLRP3-mediated macrophage pyroptosis plays a pivotal role in sepsis-induced ALI by exacerbating inflammation and organ damage. The challenge in therapeutically targeting this pathway lies in its intricate regulation within the immune system’s response to sepsis. Researches have demonstrated the therapeutic potential of modulating this pathway (Liu et al. 2022a, b; Cai et al. 2022). In addition to NLRP3-mediated macrophage pyroptosis, a recent study has also illustrated that NLRP1 is involved in the regulation of LPS-induced macrophage pyroptosis in sepsis (Li et al. 2023a, b). Nonetheless, the complex interplay of immune responses highlights the complexity of sepsis pathophysiology (Cui et al. 2023). Advancing treatment strategies for sepsis-induced lung injury thus necessitates a nuanced understanding of these molecular mechanisms within the systemic inflammatory response of sepsis.

The exploration of circRNAs in sepsis-induced lung injury has yielded critical insights into their molecular functions. Research has revealed unique circRNA expression profiles in lung tissues during sepsis, underscoring their role in modulating immune and cellular responses (Bao et al. 2019; Zou et al. 2020; Yuan et al. 2020). Specific circRNAs, such as circ-Fryl, circC3P1, circ_0001679, and circ_0001212, have been linked to protective effects against lung injury in sepsis by targeting miRNA pathways, highlighting their therapeutic potential (Zou et al. 2020; Shen et al. 2022; Jiang et al. 2020). Furthermore, previous studies have highlighted the role of circRNAs in NLRP3-mediated macrophage pyroptosis, including circ_0075723 (Yang et al. 2023), circACTR2 (Fu et al. 2022) and hsa_circ_0029589 (Guo et al. 2020). CircMAPK1, an emerging circRNA, remains largely unexplored. It has been identified as encoding a novel protein implicated in gastric cancer progression (Jiang et al. 2021). Additionally, its role in modulating immune responses in lung adenocarcinoma has been reported (Zhao et al. 2023). However, its specific functions in the context of sepsis are yet to be elucidated. Our research adds to this understanding, showing that circMAPK1 was upregulated in sepsis patients and exacerbates lung injury, primarily regulated NLRP3-mediated macrophage pyroptosis via UPF1/KDM2B/WNK1/NLRP3 pathway. Additionally, lungs are composed of multiple types of cells, such as pulmonary epithelial cell, pulmonary endothelial cells, pulmonary fibroblasts and pulmonary interstitial cells. If circMAPK1 was also expressed in these cells and participated in the regulation of biological processes merits in-depth investigation in the future study.

UPF1 is implicated in the regulation of various diseases (Staszewski et al. 2023). It acts as a key player in maintaining genetic expression fidelity by degrading aberrant mRNAs and managing protein quality (Kim and Maquat 2019). UPF1 has been identified as a RBP binding to circRPPH1 influencing tumor process (Xu et al. 2022), and recruited by circLPAR1 to modulating GDF-15 mRNA stability involved in Alzheimer’s disease (Xiong et al. 2023). However, the role of UPF1 in sepsis or macrophage pyroptosis remains undefined. Our study demonstrated that UPF1 serves as a RBP of circMAPK1 to promote KDM2B mRNA decay in sepsis-induced lung injury. This highlights a novel mechanistic pathway by which circMAPK1 exerts its influence in sepsis.

Epigenetic modifications are involved in regulating sepsis, including DNA methylation, histone modification and chromatin remodeling (Wu et al. 2023). KDM2B, an H3K36me2-specific demethylase, is implicated in the regulation of sepsis-related inflammation (Li et al. 2023a, b), yet its specific function in sepsis-induced lung injury remains to be elucidated. Our findings illustrated a decrease in KDM2B expression in patients with sepsis, and circMAPK1-recruited UPF1 modulating its mRNA stability. As a demethylase acting on H3 histones, alterations in KDM2B expression could lead to genomic dysregulation. KDM2B mediates the demethylation of H3K79me (Kang et al. 2018), H3K4me3 (Zheng et al. 2018) and H3K36me2/3 (Yan et al. 2018), with the demethylation of H3K36me2 notably influencing DNA methylation. Previous studies have demonstrated that H3K36me2 recruits DNMT3A, shaping the intergenic DNA methylation landscape (Weinberg et al. 2019). In the current study, we further validated the role of KDM2B-mediated H3K36me2 demethylation in which KDM2B mediated the enrichment of H3K36me2 at WNK1 promoter and regulated WNK1 expression. By contrast, H3K36-specific histone methyltransferase (HMTs), such as NSD1, SMYD2, SETD2 and ASH1L, mediate the methylation of H3K36 (Huang and Zhu 2018). Nevertheless, little is known about the detailed mechanism by which KDM2B or other HMTs modulated H3K36 methylation at WNK1 promoter, which would be further explored in our future study.

WNK1, a key regulator in ion homeostasis, significantly influences macrophage pyroptosis and NLRP3 inflammasome activity (Mayes-Hopfinger et al. 2021). Inhibition of WNK1 has been shown to enhance NLRP3 inflammasome activation, leading to increased pyroptosis and inflammatory cytokine production (Mayes-Hopfinger et al. 2021). In sepsis models, WNK1/TGF-β-activated kinase 1 (TAK1) axis suppresses LPS-induced inflammation and the activation of macrophages via NF-κB and MAPK signalings (Arai et al. 2020). However, the biological role of WNK1 in sepsis-induced lung injury remains elusive. Our findings demonstrated that the expression of WNK1 was downregulated in the blood samples of patients with sepsis, as well as in LPS/ATP-treated macrophages. In addition, WNK1 negatively regulated NLRP3-mediated macrophage pyroptosis. These findings advance the understanding of WNK1’s role in inflammation modulation during sepsis, deepening the insights into its potential therapeutic implications.

## Conclusion

In conclusion, our findings illustrated that circMAPK1 promoted KDM2B mRNA decay via recruiting UPF1, thereby inhibiting KDM2B-mediated WNK1 demethylation to suppress WNK1 expression, and ultimately triggering NLRP3-mediated pyroptosis to exacerbate sepsis-induced lung injury (Fig. S2). These findings contribute to a deeper understanding of sepsis pathophysiology and open up new possibilities for targeted therapeutic interventions. Future research should continue to explore the intricate molecular interactions and consider the systemic effects of sepsis to develop more effective treatments for sepsis-induced lung injury.

## Electronic supplementary material

Below is the link to the electronic supplementary material.

Supplementary Figure S1: Transcriptome sequencing of circRNAs in PBMCs of patients with septic lung injury. PBMCs of septic lung injury patients were analyzed by transcriptome sequencing. (**A**) Heatmap showed differentially expressed molecules in PBMCs. (**B**) Volcano plot showed differentially expressed molecules in PBMCs

Figure S2: Graphic abstract of this study. circMAPK1 facilitated KDM2B mRNA decay by recruiting UPF1, thereby suppressing KDM2B-WNK1 promoter association and KDM2B-mediated WNK1 demethylation. The downregulation of WNK1 thus facilitated NLRP3-mediated macrophage pyroptosis in sepsis-induced ALI

## Data Availability

The datasets generated during and/or analysed during the current study are available from the corresponding author on reasonable request.

## Abbreviations

ALI: Acute lung injury
ATP: Adenosine triphosphate
BMDMs: Bone marrow-derived macrophages
BSA: Bovine serum albumin
CCK-8: Cell counting kit-8
ChIP: Chromatin immunoprecipitation
circRNAs: Circular RNAs
CLP: Cecal ligation and puncture
ELISA: Enzyme-linked immunosorbent assay
FISH: Fluorescence in situ hybridization
H3K36me2: Dimethylation of H3K36
H&E: Hematoxylin and eosin
HRP: Horseradish peroxidase
HMTs: Histone methyltransferase
IF: Immunofluorescence
IHC: Immunohistochemistry
KDM2B: Lysine-specific demethylase 2B
LDH: Lactate dehydrogenase
LPS: Lipopolysaccharide
MPO: Myeloperoxidase
MSP: Methylation-specific PCR
PBMCs: Peripheral blood mononuclear cells
PBS: Phosphate-buffered saline
PFA: Paraformaldehyde
qRT-PCR: Quantitative real-time polymerase chain reaction
RBPs: RNA immunoprecipitation
RIP: RNA-binding proteins
SIRS: Systemic inflammatory response syndrome
TAK1: TGF-β-activated kinase 1
TdT: Terminal deoxynucleotidyl transferase
TUNEL: Terminal deoxynucleotidyl transferase dUTP nick end labeling
UPF1: Upstream frameshift 1
