# Supplementary figures and images for: CircMAPK1 induces cell pyroptosis in sepsis-induced lung injury by mediating KDM2B mRNA decay to epigenetically regulate WNK1
Source: Mol Med. 2024 Sep 19;30:155. doi: 10.1186/s10020-024-00932-6 (PMC11414303; doi:10.1186/s10020-024-00932-6)

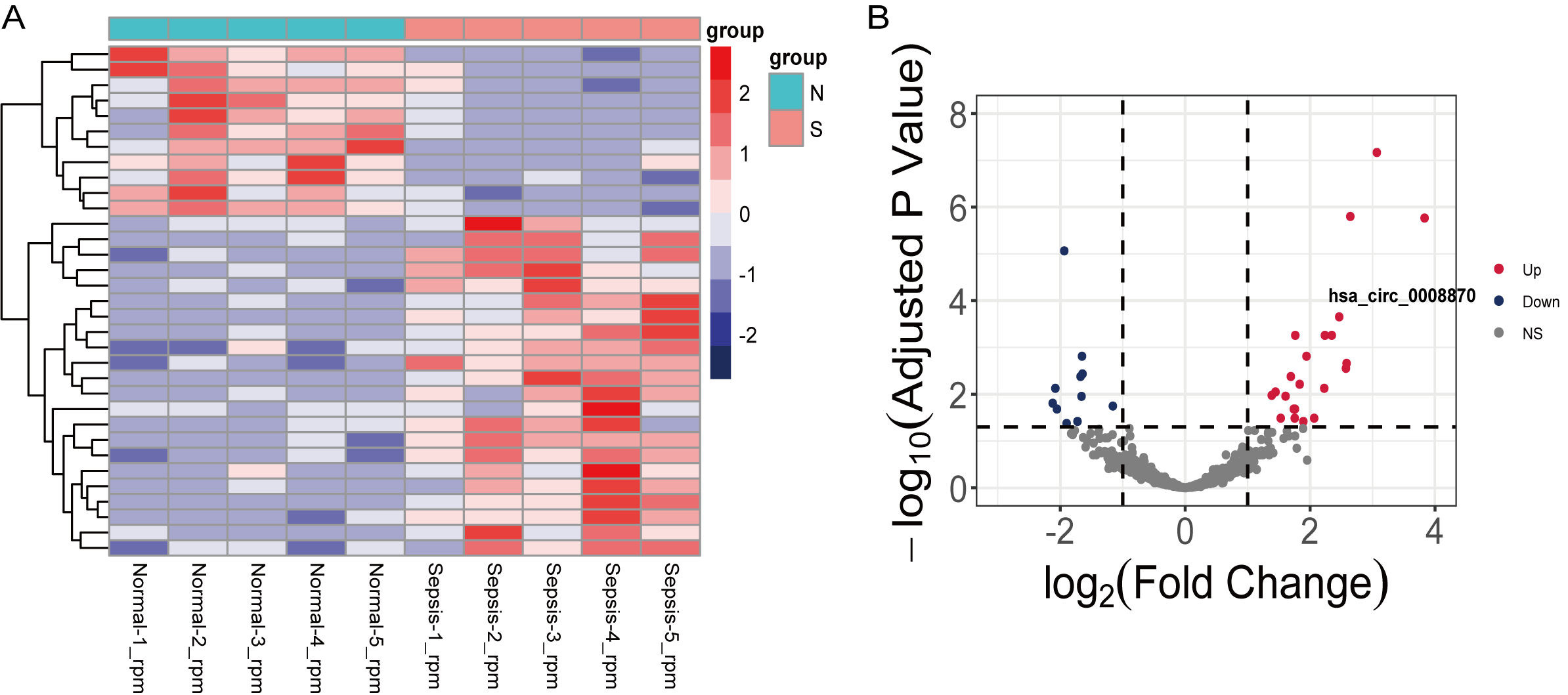

Supplement: Supplementary file 1 — Supplementary Figure S1: Transcriptome sequencing of circRNAs in PBMCs of patients with septic lung injury. PBMCs of septic lung injury patients were analyzed by transcriptome sequencing. (A) Heatmap showed differentially expressed molecules in PBMCs. (B) Volcano plot showed differentially expressed molecules in PBMCs [file 10020_2024_932_MOESM1_ESM.png]

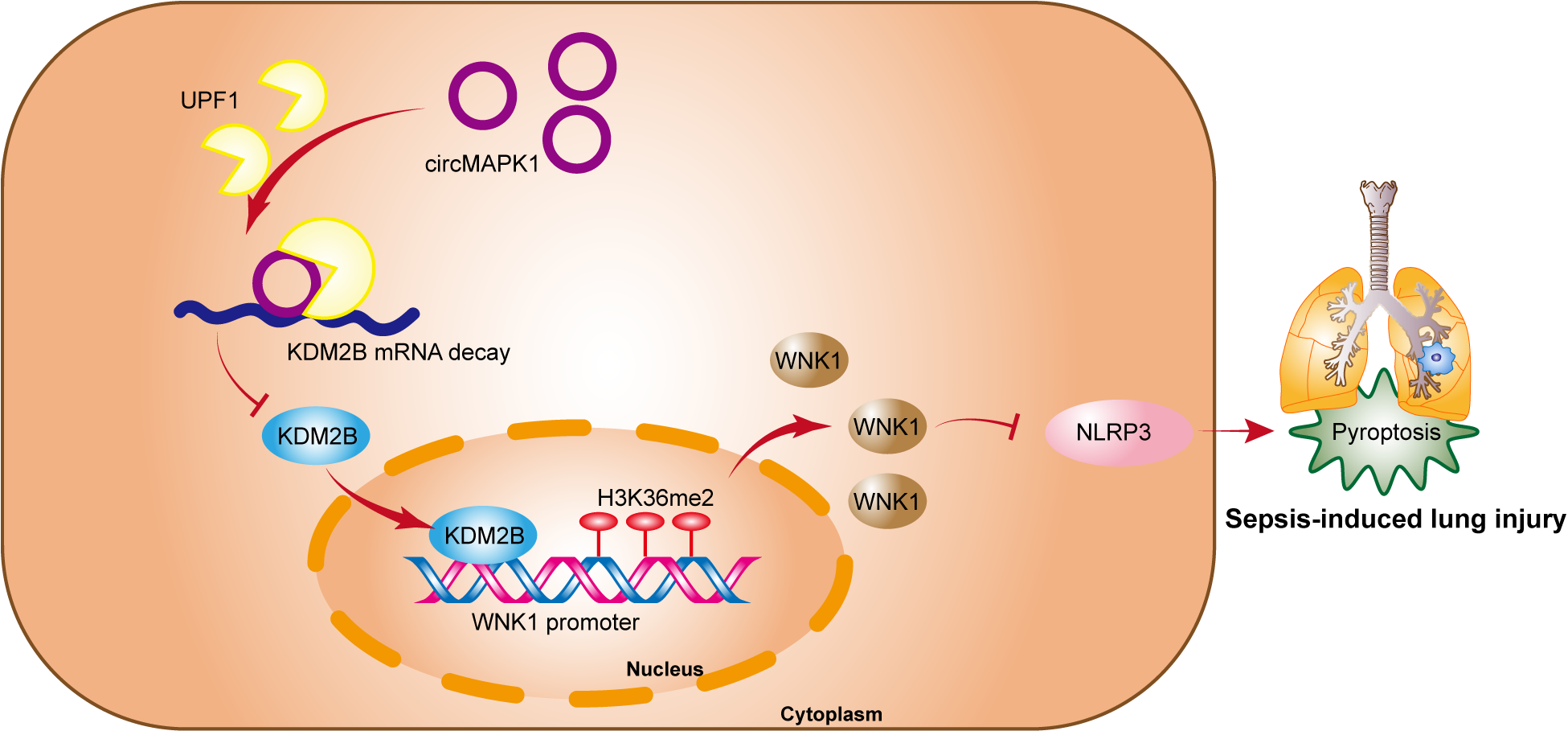

Supplement: Supplementary file 2 — Figure S2: Graphic abstract of this study. circMAPK1 facilitated KDM2B mRNA decay by recruiting UPF1, thereby suppressing KDM2B-WNK1 promoter association and KDM2B-mediated WNK1 demethylation. The downregulation of WNK1 thus facilitated NLRP3-mediated macrophage pyroptosis in sepsis-induced ALI [file 10020_2024_932_MOESM2_ESM.png]
